# Prevalence, intensity, and associated factors of soil-transmitted helminth and schistosome infections after multiple rounds of preventive chemotherapy among schoolchildren in five selected district councils in Tanzania

**DOI:** 10.1371/journal.pntd.0013310

**Published:** 2025-07-23

**Authors:** Clarer Jones, Mohamed Nyati, Abdallah Zacharia, Stephen Gabriel Mbwambo, Huda Omary, Billy Ngasala

**Affiliations:** 1 Neglected Tropical Diseases Control Program, Ministry of Health, Dodoma, Tanzania; 2 Muhimbili University of Health and Allied Sciences, Dar es salaam, Tanzania; Uniformed Services University: Uniformed Services University of the Health Sciences, UNITED STATES OF AMERICA

## Abstract

**Background:**

Schistosomiasis and soil-transmitted helminthiasis (STH) are widespread in Tanzania mainland, affecting all 184 districts. The Ministry of Health, through the National Neglected Tropical Diseases Control Program (NTDCP) has addressed these parasitic infections by administering annual preventive chemotherapy (PC) with praziquantel for schistosomiasis and albendazole for STH. This study aimed to assess the prevalence, infection intensity, and associated factors of schistosomiasis and STH among school-aged children (SAC) in five selected district councils of Tanzania after a minimum of five rounds of PC.

**Methodology/Findings:**

A cross-sectional survey was carried out in five district/town councils; Iringa District Council (DC), Nanyamba Town Council (TC), Ruangwa DC, Tanganyika DC, and Kalambo DC. We randomly selected 15 wards within each DC/TC and then chose one primary school from each ward, totaling 15 schools per district. From each school 30 children (15 boys and 15 girls) aged 10–14 years were sampled, totaling 2250 participants. Urine samples were analyzed using filtration methods to detect schistosomiasis, while stool samples were examined using the Kato-Katz method. Demographic, and water, sanitation and hygiene (WASH) data were also collected through standardized questionnaires. The prevalence of any STH infection varied across the councils, with the lowest prevalence in Iringa DC (0.7%) and highest prevalence in Tanganyika DC (7.1%). For schistosomiasis, the overall prevalence of any schistosome infection was 9.3%. Nanyamba TC reported the highest prevalence at 21.8%, while Iringa DC recorded the lowest at 2.0%.

**Conclusion:**

The study indicates that STH prevalence remains relatively low (< 10%) in the majority of surveyed districts, however four out of five surveyed districts require distribution of PC once per two years for five years. For schistosomiasis, all district had at least one ward that require annual distribution of PC for the entire population (aged ≥2 years) for 5 years.

## Author summary

Schistosomiasis and soil transmitted helminthiasis (STH) are parasitic diseases affecting children across Tanzania. The Tanzanian Ministry of Health addresses these infections by providing annual preventive medications to school-aged children through the National Neglected Tropical Diseases Control Program (NTDCP). After five or more years of treatment, the World Health Organization (WHO) recommends evaluating program effectiveness to address remaining infection challenges. This study assessed infection prevalences and related factors for schistosomiasis and STH among children in five selected districts. The study included 2,250 children from 75 schools, each provided urine and stool samples. Researchers also collected data on children’s living conditions, hygiene practices, and water access. The results showed the prevalence of any STH infection was 3.4%, with the highest prevalence observed in Tanganyika DC (7.1%), and lowest prevalence in Iringa DC (0.7%). Based on WHO decision tree for determining the frequency of PC distribution for STH, four out of five districts which fall in the category of prevalence between 2 to <10.0%, will require annual distribution of PC for 5 years. For schistosomiasis the overall prevalence of any schistosome infection was 9.3%. Nanyamba TC reported the highest prevalence at 21.8%, while Iringa DC recorded the lowest at 2.0%. According to WHO decision tree for determining the frequency of PC distribution and impact assessment for schistosomiasis all surveyed districts will require annual distribution of PC for the entire population (aged ≥2 years) for 5 years.

## 1. Introduction

Schistosomiasis and soil transmitted helminthiasis (STH) are two of the most prevalent neglected tropical diseases (NTDs), particularly in low- and middle-income countries. These diseases thrive in environments where access to clean water, sanitation, and hygiene is limited, disproportionately affecting impoverished populations [1]. Schistosomiasis is caused by parasitic flatworms of the genus *Schistosoma*, which enter the human body through contact with contaminated water. This waterborne disease is endemic in 78 countries, placing over 700 million people at risk [2]. Approximately 251.4 million individuals reside in areas with moderate to high transmission rates [3]. Furthermore, schistosomiasis affects over 290 million people worldwide, with around 93% of infections occurring in sub-Saharan Africa [4,5]. Children, especially school-aged children (SAC, typically aged 5 – 14 years), are particularly vulnerable due to activities such as swimming and fishing in infested waters [6].

Among infected individuals, an estimated 123 million are children, making this group the most affected in terms of both prevalence and intensity of infection [6]. Schistosomiasis in children has been associated with a range of health issues, including malnutrition, impaired cognitive development, iron-deficiency anemia, and poor academic performance. Left untreated, it can lead to more severe complications such as hepatosplenic disease, which can have long-term consequences for both individual health and public health systems [7]. Recognizing this, the World Health Assembly (WHA) passed resolution 54.19, urging member states to treat at least 75% of SAC who are at risk of morbidity from schistosomiasis [8].

Soil-transmitted helminthiasis are another group of parasitic infections prevalent in Tanzania and other tropical and subtropical regions. The infections are caused by three helminthic nematodes, namely *Ascaris lumbricoides*, *Trichiuris trichiura*, and hookworms (*Ancylostoma duodenale* and *Necator americanus*). STH-related infections occur primarily through the fecal-oral route, with individuals ingesting infective eggs of *A. lumbricoides* and *T. trichiura* or through skin penetration by hookworm filariform larvae. Globally, approximately 1.5 billion people are affected by STH, with the highest prevalence occurring in sub-Saharan Africa, China, and South America. Similar to schistosomiasis, SAC are the most affected group [9–11]. In Tanzania, the most recent estimates suggest that the prevalence of STH ranges between 20% and 50% in various regions, with the northwestern areas near Lake Victoria reporting the highest prevalences [12]. Like schistosomiasis, STH infections are associated with poor health outcomes in SAC, including malnutrition and impaired cognitive development [10].

The global strategy for schistosomiasis and STH control, as recommended by the World Health Organization (WHO), primarily relies on preventive chemotherapy (PC) using praziquantel for schistosomiasis [13] and albendazole for STH [11]. The goal is to control morbidity and eventually eliminate schistosomiasis and STH as a public health problem [14]. WHO has outlined three key recommendations for treatment depending on the prevalence of *Schistosoma* infections within a community. For communities where prevalence is equal to or exceeds 10%, annual PC is recommended, with a target treatment coverage of at least 75% for all age groups from two years old, including pregnant women (after the first trimester) and lactating women. For communities with a prevalence below 10%, treatment strategies may vary based on programmatic objectives and available resources, which may involve continuing PC or adopting a clinical test-and-treat approach. In communities where annual PC has not yielded satisfactory results despite adequate coverage, WHO suggests biannual PC as an alternative to annual treatment [4,13]. In the case of STH, in treatment-naïve communities, biannual PC is recommended when baseline-estimated prevalence exceeds 50%, annual PC is advised when prevalence is between 20–49.9%, and clinical case management is suggested when prevalence is below 20% [11].

In sub-Saharan Africa, including Tanzania, PC with praziquantel and albendazole has been widely adopted, primarily through school- or community-based platforms targeting districts with moderate to high schistosomiasis and STH prevalence [15]. Consequently, the epidemiology of schistosomiasis and STH has changed, prompting the need to reassess infection prevalences post-treatment [16]. The Expanded Special Project for Elimination of Neglected Tropical Diseases (ESPEN) has recommended that countries conducting at least five years of effective PC reassess prevalence to determine if the frequency of treatment should be modified. Notably, the purpose of these impact assessments is not to measure changes in prevalence but rather to determine whether the current prevalence still meets the target thresholds, particularly the 2%, 10% and 20% prevalence mark [17].

Despite the clear recommendation for impact assessments, WHO has not provided evidence-based guidance on how these surveys should be conducted. This presents challenges, especially in countries like Tanzania, where schistosomiasis and STH programs are shifting from district-level PC to sub-district or ward-level interventions to better target areas with high transmission. In Tanzania, two species of *Schistosoma* are prevalent: *S. mansoni*, which causes intestinal schistosomiasis, and *S. haematobium*, which leads to urogenital schistosomiasis. Historically, *S. mansoni* has been more prevalent in regions surrounding Lake Victoria, while *S. haematobium* is more widespread in inland areas, particularly along the southern shores of Lake Victoria [2]. The three STH parasites are endemic throughout the country. However, recent data on the distribution of both schistosomiasis and STH parasites are limited, highlighting the need for updated prevalence surveys [18,19].

Tanzania has adopted a PC strategy using praziquantel for schistosomiasis and albendazole for STH, primarily targeting SAC. All 184 districts in Tanzania are endemic for schistosomiasis and STH, and have implemented at least five rounds of PC. Among these, 19 districts have been classified as high-prevalence areas for schistosomiasis and receive yearly treatment, while the remaining 165 districts, with moderate prevalence, conduct treatment every two years. However, due to resource constraints, high-risk adults are not consistently targeted for schistosomiasis treatment [19].

Efforts to control schistosomiasis and STH through PC have led to significant reductions in prevalence and infection intensity. Survey data collected between 2016 and 2022 indicate a decline in moderate and high-endemicity wards. Precision mapping surveys conducted in 2018 further confirmed that many wards no longer required PC for schistosomiasis. In 2022, mapping efforts continued in 53 districts, identifying areas that remain endemic and those that have achieved lower prevalence. These findings are crucial for guiding future control efforts and resource allocation. Despite progress, several challenges remain. There is limited information on the current post-PC geographical distribution and intensity of schistosomiasis and STH in many districts, raising questions about where intensive interventions are still needed and where treatment can be reduced [19]. Understanding the current epidemiology of these infections is vital for informed decision-making, especially as Tanzania moves from morbidity control to elimination of schistosomiasis and STH as public health problems [15,20].

To address these gaps, the Tanzania National Neglected Tropical Diseases Control Program (NTDCP), in collaboration with international partners, conducted assessments in five implementation units across the country. This initiative aimed to generate updated data on infection prevalence and identify risk factors, which will inform future PC strategies [21]. As of 2023, 82 district councils in Tanzania have been remapped, but 102 councils still require impact surveys [18]. The data generated from these efforts are critical for the ongoing fight against schistosomiasis and STH in Tanzania, ensuring that resources are targeted where they are most needed. The current study presents the findings of the schistosomiasis and STH impact assessment survey that has been conducted in five implementation units following multiple rounds of PC among SAC in Tanzania.

## 2. Methodology

### 2.1. Study area

**Fig 1:**
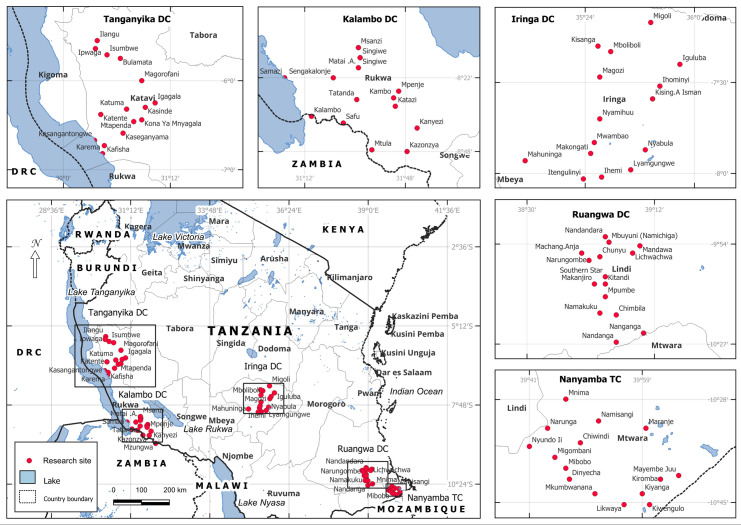
Maps of selected study wards in Tanganyika DC, Iringa DC, Kalambo DC, Ruangwa DC and Nanyamba TC) QGIS Ver. 3.28—CC-BY license 4.0.). The layers are from https://gadm.org/download_country.html and those from OpenStreetMap (OSM) are freely accessible from https://www.openstreetmap.org/#map=6/-6.40/35.00 and can be shared under CC-BY license 4.0.

This study was conducted across five district councils: Nanyamba Town Council (TC) in Mtwara Region, Ruangwa District Council (DC) in Lindi Region, Iringa DC in Iringa Region, Kalambo DC in Rukwa Region, and Tanganyika DC in Katavi Region (Fig 1). These areas are co-endemic for both schistosomiasis and STH. A baseline survey conducted in 2004 reported schistosomiasis prevalence at the ward level ranging from 22.3% to 33.0% in Nanyamba TC, 16.9% to 56.5% in Ruangwa DC, 5.2% to 25.0% in Iringa DC, 4.0% to 100.0% in Mpanda DC and 6.2% to 50.5% in Kalambo DC. Since 2011, these councils have implemented more than five rounds of PC with praziquantel and albendazole, achieving at least five effective rounds with coverage of ≥ 75%, making them eligible for an impact assessment. For schistosomiasis, Kalambo DC had minimum number of school MDA rounds (six) while Ruangwa DC had the maximum number of school MDA rounds (twelve). For STH, both school and community MDA were implemented. For school MDA, Iringa had the minimum rounds (nine) while Ruangwa DC and Tanganyika TC had the maximum rounds of MDA (twelve). For community MDA, the minimum MDA were implemented in Kalambo DC and Iringa DC (five) while Ruangwa had maximum number of rounds (twelve). Since the implementation of MDA, no any survey was conducted on the selected councils to assess the disease status.

### 2.2. Study design

This was school-based, cross-sectional study conducted in May 2024 to determine the prevalence, intensity, and associated factors of schistosomiasis and STH following multiple rounds of praziquantel and albendazole PC among SAC in five selected district councils in Tanzania.

### 2.3. Sample size

A sample size of 30 SAC per primary school was set to represent the target number of children who provided valid urine and stool specimens. Additionally, 6 extra SAC (3 boys and 3 girls) were selected during the randomization process as alternates. These alternates were asked to participate in the study and provide samples in the event that any of the initially selected 30 SAC were unable or unwilling to do so. Therefore, in each primary school, a total of 36 SAC (18 boys and 18 girls) were selected to ensure the required sample size was met.

### 2.4. Sampling techniques

We applied the approach recommended by the outcomes of the Schistosomiasis Oversampling Study (SOS). The major goal of the SOS was to identify optimal survey sampling methods for conducting impact assessments that are feasible for country programs, cost-effective and result in appropriate treatment classifications. In this approach, a two-step impact assessment strategy is used (MEETING REPORT Schistosomiasis Oversampling Study: Survey Strategy Selection Nairobi, Kenya - May 18 &19, 2023 (https://www.eliminateschisto.org › sites › gsa › files... · PDF file).

The sampling procedure was conducted in three steps. First, the number of primary schools per ward was determined to ensure good geographical representation. The 15 primary schools were selected through systematic random sampling using the Practical Assessment Systematic Sampling Tool. Census data provided a list of all wards in each district, which were ordered by location: East to West for Nanyamba TC, South to North for Mpanda DC, Ruangwa DC, and Iringa DC, and Southwest to Northwest for Kalambo DC. This list was used to select wards and primary schools to include in the survey.

Second, primary schools within selected wards were chosen randomly when there was more than one school. Schools were numbered, and one was randomly selected using slips of paper drawn from a hat. The list of schools, both governmental and non-governmental, was obtained from the President’s Office, Regional Administration, and Local Government (PO-RALG). Schools without students in grades 4–6 were excluded from the list.

Finally, SAC were selected randomly from age-based groups (10–14 years old) in separate lines for boys and girls, with 15z students of each sex sampled.

### 2.5. Eligibility criteria

Eligible participants consisted of all SAC aged 10–14 years, enrolled in grades 4–6, permanent residents of the selected areas (at least lived in the area for > 2 years), and no history of taking anthelminthic drugs for the past six months and agreed to provide urine and stool samples. Children presenting with illness, such as fever, were excluded from the survey and referred to healthcare providers for appropriate care. Additionally, children whose parents declined consent for their participation were not included in the study.

### 2.6. Data collection procedures

#### 2.6.1. Questionnaire interview.

The questionnaire used for the survey was adapted from the WHO ESPEN Collect tool. A centralized master database was maintained for data entry, cleaning, and quality assurance. Three types of questionnaires were administered via Android devices: (i) a school-level form, (ii) an individual questionnaire, and (iii) a laboratory results form. All forms had been previously validated and utilized in similar epidemiological surveys within the NTD program. The school form gathered information on country name, sub-district (ward name), school names, geographic coordinates, enrollment numbers, and the availability of water, sanitation, and hygiene (WASH) facilities and school participation in PC campaigns. The individual questionnaire captured demographic data, including age, sex, grade, time of residence, WASH activities, and participation in PC campaigns. The laboratory forms documented the laboratory findings from processed urine and stool samples.

#### 2.6.2. Stool and urine samples collection.

Stool and urine samples were collected from each of the selected SAC. Children were provided with instructions on how to hygienically collect urine and stool sample and they were given tissue paper and pre-labeled wide-mouthed containers (bearing identification numbers) for stool and urine collection. Sample collection occurred between 10:00 AM and 2:00 PM. Children were instructed to collect 20 g of fresh stool and 20 mL of urine. Upon receipt, both types of samples were logged in a sample registration form for proper tracking.

#### 2.6.3. Parasitological procedures.

***Stool processing and examination*:** Stool samples were processed to detect the presence and intensity of *S. mansoni* and STH infections using the double slides Kato-Katz method. In brief, stool samples were homogenized, and a small portion was placed on a paper and sieved using nylon mesh. The sieved stool was transferred to a hole of template placed on a pre-labeled glass slide. After filling the hole with the fecal material and removing the excess stool, the template was removed and a cellophane strip soaked in methylene blue solution was placed on top of the measured stool sample. The slide was inverted and firmly pressed to spread the stool evenly.

Microscope slides were examined immediately after preparation to identify hookworm eggs, which clear rapidly. Technologists scanned the entire smear systematically using a “zig-zag” pattern, counting eggs using a hand tally counter. Data on the number and type of eggs were recorded, with a notation of “0” if no eggs were observed [22]. During day two, the slides were reexamined for of *S. mansoni* and other STH. Twenty percent of slides were randomly selected for quality control, while the remaining slides were disposed of appropriately.

***Urine processing and examination*:** The urine filtration technique was employed to detect and quantify *S. haematobium* eggs. Briefly, urine sample was thoroughly mixed, and a 10 mL aliquot was drawn into a syringe. A filter unit was then attached, and the syringe was held vertically while the plunger was pressed to pass the urine through the filter into a container. The syringe was carefully removed, and air was drawn into the syringe, which was reconnected to the filter unit and expelled to ensure that all excess urine was removed, leaving the eggs adhered to the filter. The filter unit was then unscrewed, and the filter was carefully placed onto a glass microscope slide using tweezers. A drop of Lugol’s iodine was added to the filter to stain the eggs, and after 15 seconds, the slide was examined under the microscope to detect the presence of *Schistosome* eggs. The number of eggs was recorded per 10 mL of urine [22].

### 2.7. Data analysis

Data were entered in Open Data Kit (ODK) collect. Data cleaning and analysis were performed using Stata Version 17 (StataCorp, 2017, College Station, TX). Descriptive and inferential analyses were conducted on both continuous and categorical variables. Continuous variables were summarized using means and standard deviations, while categorical data were presented as frequency counts and percentages (proportions). For *S. mansoni* and STH, arithmetic mean egg counts were calculated based on two Kato-Katz smears (slides), and multiplied by 24 to estimate individual eggs per gram (epg) of feces. Infection intensity for *S. mansoni* was classified according to the WHO criteria as low (1–99 epg), moderate (100–399 epg), and heavy (≥ 400 epg). For *S. haematobium*, geometric mean egg output was calculated from infected children, with intensity of infection classified as light (<50 eggs/10 mL urine) and heavy (≥ 50 eggs/10 mL urine). For STH, the intensity was categorized as light (0–1999 epg), moderate (2000–3999 epg), and heavy (≥ 4000 epg) for hookworm; light (0–4999 epg), moderate (5000–49999 epg), and heavy (≥ 50000 epg) for *A. lumbricoides*; and light (0–999 epg), moderate (1000–9999 epg), and heavy (≥ 10000 epg) for *T. trichiura*.

To account for the hierarchical structure of the data, where schools were nested within districts, a mixed-effects logistic regression model was employed. This model included random intercepts for schools and districts to capture potential clustering effects. All statistical analyses were conducted using STATA (version 17; Stata Corp, College Station, TX, USA) and R Statistical Software (version 3.4.3, https://www.r-project.org/) were used for graphical analysis. The melogit command in Stata was used to fit the mixed effects model.

### 2.8. Ethics statement

Ethical approval for the study was obtained from the Muhimbili University of Health and Allied Sciences Ethical Review Board prior to data collection. Permission to conduct the survey was granted by the Regional and District Administrative Authorities of the relevant regions and districts. Written informed consent was secured from head teachers and parents or guardians, while children aged 10–14 years provided written assent. The purpose of the survey, including participants’ rights to participate or withdraw, as well as potential risks and benefits, was thoroughly explained to both the participants and their guardians. To ensure confidentiality, participants were assigned unique codes, which were used for identification purposes instead of their names. All survey records were securely stored by the principal investigator.

## 3. Results

### 3.1. Demographic characteristics of study participants

A total of 75 schools were included in this study, with 15 schools from each DC or TC (Iringa DC, Kalambo DC, Nanyamba TC, Ruangwa DC, and Tanganyika DC). In total, 2250 students participated, with 30 students selected from each school and 450 from each district. Overall, 49.5% of the students were male, and 50.5% were female, the gender distribution was very balanced across all districts with almost 50–50 ratio between male and female students. The highest proportion of students, 27.2%, were aged 12 years, with some variation across districts. Iringa had a lower proportion of students in this age group (22.0%), while Ruangwa had a higher proportion (31.6%). Overall, Grade VI had the largest proportion of students 775 (34.4%), with values ranging from 148 (32.9%) in Kalambo to 177 (39.3%) in Nanyamba (Table 1).

**Table 1:** Demographic characteristics of school children in the 75 surveyed schools.

Parameter	Overall	Districts				
		Iringa, n (%)	Kalambo n (%)	Nanyamba n (%)	Ruangwa n (%)	Tanganyika n (%)
Sex						
Male	1114 (49.5)	220 (48.9)	220 (48.9)	227 (50.4)	224 (49.8)	223 (49.6)
Female	1136 (50.5)	230 (51.1)	230 (51.1)	223 (49.6)	226 (50.2)	227 (50.4)
Age (years)						
10	534 (23.7)	187 (41.6)	99 (22)	91 (20.2)	102 (22.7)	55 (12.2)
11	556 (24.7)	128 (28.4)	111 (24.7)	113 (25.1)	117 (26)	87 (19.3)
12	612 (27.2)	99 (22)	124 (27.6)	142 (31.6)	126 (28)	121 (26.9)
13	367 (16.3)	31 (6.9)	72 (16)	74 (16.4)	73 (16.2)	117 (26)
14	181 (8)	5 (1.1)	44 (9.8)	30 (6.7)	32 (7.1)	70 (15.6)
Grade of the student						
IV	757 (33.6)	124 (27.6)	162 (36)	140 (31.1)	155 (34.4)	176 (39.1)
V	718 (31.9)	161 (35.8)	140 (31.1)	133 (29.6)	142 (31.6)	142 (31.6)
VI	775 (34.4)	165 (36.7)	148 (32.9)	177 (39.3)	153 (34.0)	132 (29.3)

### 3.2. Prevalence of soil-transmitted helminths

**Fig 2:**
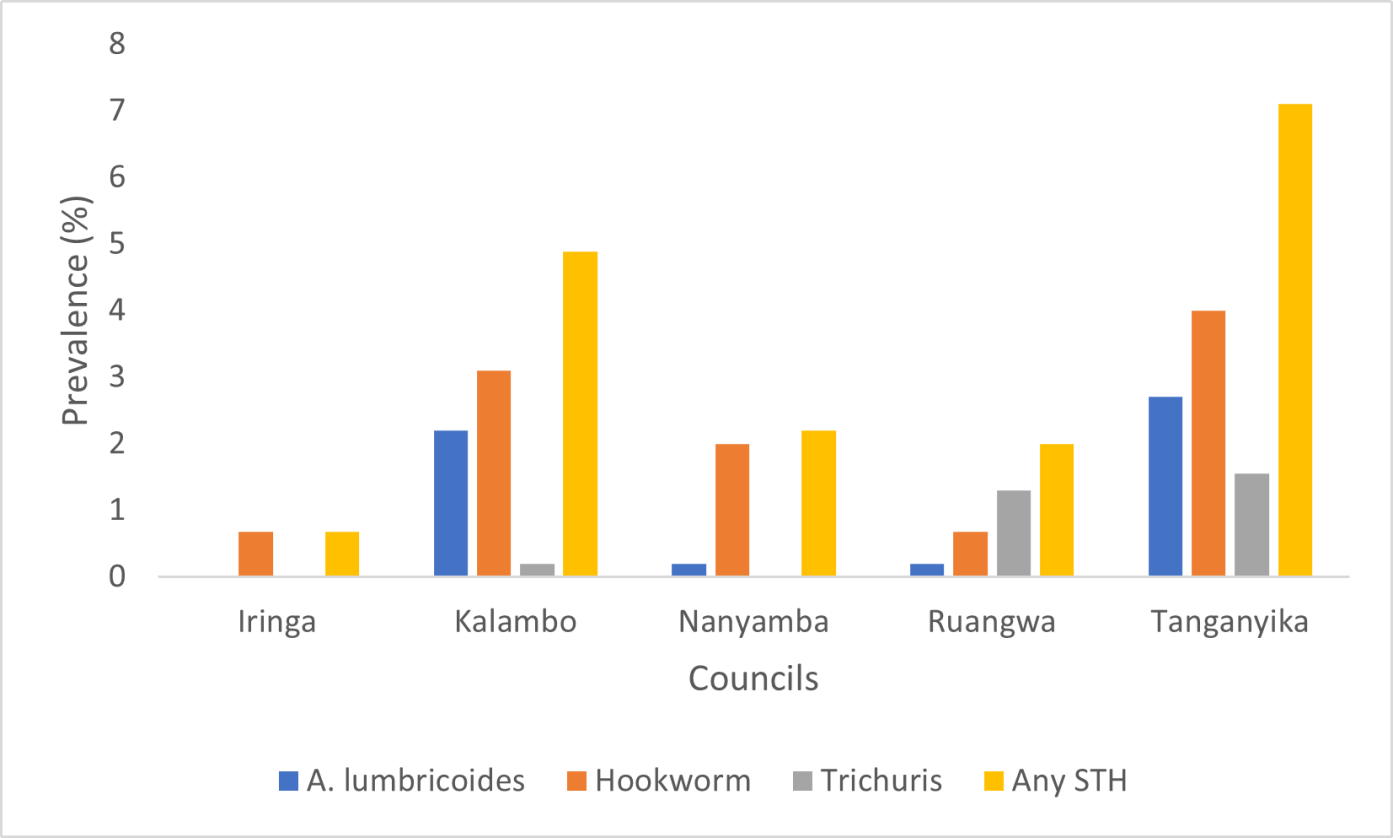
Prevalence of Soil transmitted helminths in the five surveyed councils.

The overall prevalence of hookworm was 2.1%, *A. lumbricoides* was 1.1%, and *T. trichiura* was 0.6%. Nine students (0.4%) were co-infected with two species. Of these, five students had both hookworm and *A. lumbricoides*, two had hookworm and *T. trichiura*, and two had *A. lumbricoides* and *T. trichiura*. The prevalence of any helminth species was 3.4%. Other species were detected in three students (0.1%), with two having *H. nana* and one having *Enterobius vermicularis*. Hookworm 2.2% and *A. lumbricoides* 1.5% infections were slightly higher among males compared to females. *T. trichiura* had an equal prevalence among both males and females 0.6% each (Table 2). The prevalence of hookworm varied across councils: 0.7% in Iringa DC, 3.1% in Kalambo DC, 2.0% in Nanyamba TC, 0.7% in Ruangwa DC, and 4.0% in Tanganyika DC. For *A. lumbricoides*, the prevalences were 2.2% in Kalambo DC, 0.2% in Nanyamba TC, 0.2% in Ruangwa DC, and 2.7% in Tanganyika DC. *T. trichiura* was detected in 0.2% of students in Kalambo DC, 1.3% in Ruangwa DC, and 1.6% in Tanganyika DC. Tanganyika DC exhibited the highest overall prevalence of STH across all species (Fig 2).

**Table 2:** Prevalence of soil transmitted helminthiasis infection by demographics.

Variable	Soil transmitted helminthiasis infection		
	Hookworm	*A. lumbricoides*	*T. trichiura*
Sex			
Male	24 (2.2)	17 (1.5)	7 (0.6)
Female	23 (2.0)	7 (0.6)	7 (0.6)
Age (years)			
10	5 (0.9)	5 (0.9)	2 (0.4)
11	13 (2.3)	3 (0.5)	4 (0.7)
12	9 (1.5)	7 (1.1)	2 (0.3)
13	15 (4.1)	7 (1.9)	4 (1.1)
14	5 (2.8)	2 (1.1)	2 (1.1)

Hookworm 2.2% and *A. lumbricoides* 1.5% infections were slightly higher among males compared to females. *T. trichiura* had an equal prevalence among both males and females 0.6% each. The prevalence of all three species was highest among children aged 13 years; Hookworm (4.1%), *A. lumbricoides* (1.9) *and T. trichiura* (1.1) (Table 2).

Table 3 provides a classification of prevalence for STH in the surveyed districts based on WHO decision tree for determining the frequency of PC distribution and impact assessment for STH. Four out of five districts fall in the category of prevalence between 2 to <10.0%, while the remaining on fall in <2% prevalence category.

**Table 3:** Distribution of districts based on WHO prevalence threshold for PC intervention for STH.

Districts	Soil-transmitted helminths prevalence thresholds			
	<2%	2- < 10.0%	≥10- < 20%	≥20%
Tanganyika		✔		
Iringa	✔			
Kalambo		✔		
Nanyamba		✔		
Ruangwa		✔		

✔ District falling under the respective threshold.

### 3.3. Intensity of soil-transmitted helminths infection

The mean egg intensity (standard deviation [SD]) among students infected with hookworm was 82 (110) epg. For *A. lumbricoides*, the mean intensity of infection was 146 (289) epg, while for *T. trichiura*, it was 59 (58) epg. Table 3 presents the intensity of STH infections stratified by various demographic characteristics. The infection intensity for all three STH species was higher in females than males, although these differences were not statistically significant. The highest intensity of *A. lumbricoides* and *T. trichiura* infections was observed in children aged 11, whereas hookworm intensity was highest in children aged 13. None of these differences were statistically significant. Among children who did not receive albendazole, the mean hookworm intensity was 264 epg, compared to 70 epg in those who received the drug (p = 0.0372). For *T. trichiura*, the mean intensity was higher in children who did not receive albendazole (618 epg) compared to those who did (103 epg), though this difference was not statistically significant (p = 0.0565) (Table 4). When infection intensity was categorized by the WHO thresholds for classifying the intensity of STH infections, all infected students were classified as having light-intensity infections, regardless of the STH species.

**Table 4:** Mean intensity of soil-transmitted helminths infection.

Variable	Category	Soil-transmitted helminthiasis, mean (SD)		
		Hookworm	*A. lumbricoides*	*T. trichiura*
Sex	Male	72 (90.4)	114.4 (222.8)	54.9 (40.3)
	Female	92.3 (128.6)	221.1 (423.5)	63.4 (74.8)
Age (years)	10	36 (19)	28.8 (37.6)	24 (17)
	11	72.9 (59.5)	308 (360.9)	81 (99.1)
	12	44 (19.9)	298.3 (454.9)	42 (8.5)
	13	139.2 (174.6)	36 (18.3)	69 (43.1)
	14	48 (29.4)	42 (25.5)	48 (50.9)
ALB uptakeA	Yes	69.5 (86.6)	102.5 (197.2)	45 (34)
	No	264 (252.9)	618 (789.1)	144 (118.8)

### 3.4. Prevalence of schistosomiasis

The overall prevalence of schistosome infection was 9.3%, with notable variation across districts. Nanyamba TC reported the highest prevalence at 21.8%, while Iringa DC recorded the lowest at 2.0%. The overall prevalence of *S. mansoni* was 1.2%, whereas *S. haematobium* accounted for 8.3%. Tanganyika DC had the highest prevalence of *S. mansoni* at 4.9%. In contrast, Nanyamba TC exhibited the highest prevalence of *S. haematobium* (21.8%), followed by Ruangwa DC at 11.3%. At the ward level, schistosomiasis prevalence ranged from 3.3% to 63.3% in Nanyamba TC, 0.0% to 36.7% in Ruangwa DC, 0.0% to 16.7% in Iringa DC, 0.0% to 3.3% in Tanganyika DC, and 0.0% to 10.0% in Kalambo DC.

For *S. haematobium*, the prevalence was higher in male students (10.4%), aged 12 years (11.1%), and those received praziquantel (8.7%). For *S. mansoni* the prevalence was higher among male student (1.5%) and those aged 13 years (1.6%) (Table 5).

**Table 5:** Prevalence of schistosomiasis by student characteristics.

Variable	Schistosomiasis	
	*S. mansoni*	*S. haematobium*
Sex		
Male	17 (1.5)	116 (10.4)
Female	10 (0.9)	70 (6.2)
Age (years)		
10	6 (1.1)	32 (6)
11	7 (1.3)	35 (6.3)
12	6 (1.0)	68 (11.1)
13	6 (1.6)	32 (8.7)
14	2 (1.1)	19 (10.5)

Table 6 provides a classification of prevalence for schistosomiasis in the surveyed districts based on WHO decision tree for determining the frequency of PC distribution and impact assessment. All districts had at least one ward with the prevalence of schistosomiasis ≥10%. Nanyamba TC had the highest number of wards 9/15, with schistosomiasis prevalence ≥10%, while Kalambo DC had highest number of wards with schistosome prevalence <10%.

**Table 6:** Distribution of wards based on WHO prevalence threshold for PC intervention for schistosomiasis.

Districts	Number of wards with schistosome prevalence <10%	Number of wards with schistosome prevalence ≥10%
Tanganyika	10	5
Iringa	13	2
Kalambo	14	1
Nanyamba	6	9
Ruangwa	9	6

### 3.5. Intensity of schistosomiasis infection

In terms of gender, both *S. mansoni* and *S. haematobium* infections appear similar between males and females. However, *S. mansoni* intensity is higher, with males showing a mean intensity of 180.7 epg and females 187.2 epg. Across age groups (10–14 years), *S. mansoni* infection peaks at age 12 with a mean of 292, while *S. haematobium* remains more consistent, although wide ranges indicate variability in infection intensity (Table 7).

**Table 7:** Intensity of schistosomiasis infection by students’ demographics.

Variable	Schistosomiasis	
	*S. mansoni (egg/g feces)*	*S. haematobium (eggs/10ml urine)*
**Sex**		
Male	180.7 (259.4)	36.6 (26.2, 51.3)
Female	187.2 (179.5)	28.4 (19, 42.5)
**Age**		
10	136 (118.5)	27.0 (12.8, 56.8)
11	181.7 (136.7)	26.8 (15.7, 45.7)
12	292 (421.9)	40 (25.4, 63)
13	150 (193.3)	35.9 (19.2, 67.2)
14	102 (59.4)	32.3 (16.8, 61.9)

When infection intensity was classified using WHO thresholds for schistosome infection intensity, all surveyed districts had at least one ward with a prevalence of heavy-intensity infection below 1%. Nanyamba DC recorded the highest number of wards (9 out of 15) with a heavy-intensity infection prevalence of less than 1%. In contrast, Iringa DC and Kalambo DC each had only one ward with a 1% prevalence of heavy-intensity infection (Table 8).

**Table 8:** Present number of wards with <1% prevalence of heavy intensity of schistosomiasis infection.

Districts	Number of wards with <1% prevalence of heavy intensity of infections
Tanganyika	4
Iringa	1
Kalambo	1
Nanyamba	11
Ruangwa	9

### 3.6. Predictors of schistosomiasis and soil-transmitted helminths infection

For STH infection, no covariate was found significant in univariate and multivariate analysis (Table 9). Table 10 presents the mixed effect logistic regression results for factors associated with schistosomiasis infection. The multivariate model results indicated that males had 93% higher odds of schistosomiasis compared to females (OR = 1.93, 95% CI 1.38 – 2.69). Students who did bath, swim or play in nearby rivers, streams or ponds have about 65% significantly higher odds of getting schistosomiasis compared to students who did not (OR = 1.65, 95% CI 1.11 – 2.47). Univariate analysis showed that students who fished in nearby rivers, streams, or ponds had 79% higher risk of schistosomiasis compared to those who did not (OR = 1.79, 95% CI 1.18 – 2.7) but this was not statistically significant in the multivariate analysis.

**Table 9:** Results of univariate mixed model effect for factors associated with STH infection.

Parameter	Number of participants (%)	Infected (%)	Univariate		
			Odds ratio (SE)	95% CI	p-value
Sex					
Female	1136 (50.5)	33 (2.9)	Ref		
Male	1114 (49.5)	43 (3.9)	1.35 (0.33)	0.84 - 2.2	0.218
Age (years)			1.13 (0.12)	0.92 -1.38	0.243
Age group (years)					
10	534 (23.7)	11 (2.0)	Ref		
11	556 (24.7)	19 (3.4)	1.61 (0.65)	0.73 - 3.54	0.236
12	612 (27.2)	17 (2.8)	1.24 (0.51)	0.56 - 2.79	0.596
13	367 (16.3)	21 (5.7)	2.08 (0.85)	0.93 - 4.65	0.076
14	181 (8.0)	8 (4.4)	1.49 (0.77)	0.54 - 4.08	0.442
Did you receive deworming treatment with Albendazole?					
No	209 (9.3)	6 (2.9)	Ref		
Yes	2041 (90.7)	70 (3.4)	1.68 (0.9)	0.59 - 4.82	0.332
Do you use improved water source?					
Yes	1710 (76.0)	52 (3.0)	Ref		
No	540 (24.0)	24 (4.4)	1.83 (1.07)	0.59 - 5.73	0.298
Time it takes to get hand washing water and come back					
0 minutes	1200 (53.3)	50 (4.2)	Ref		
< 30 minutes	510 (22.7)	13 (2.6)	0.53 (0.32)	0.16 - 1.75	0.296
≥ 30 minutes	540 (24.0)	13 (2.4)	0.35 (0.23)	0.1 - 1.24	0.103
Do you use improved hand washing system?					
Yes	1710 (76.0)	52 (3.0)	Ref		
No	540 (24.0)	24 (4.4)	1.83 (1.07)	0.59 - 5.73	0.298
Time it takes to get hand washing water and come back					
0 minutes	1230 (54.7)	50 (4.1)	Ref		
< 30 minutes	510 (22.7)	13 (2.6)	0.56 (0.35)	0.17 - 1.89	0.354
≥ 30 minutes	510 (22.7)	13 (2.6)	0.43 (0.29)	0.12 - 1.57	0.204
Hand washing facilities available at school					
No any handwashing facility	210 (9.3)	1 (0.5)	Ref		
Water only	1680 (74.7)	67 (4.0)	5.61 (7.88)	0.36 - 88.01	0.22
Water and soap/ashes	360 (16.0)	8 (2.2)	2.6 (4.13)	0.12 - 58.19	0.546
Type of latrine available at school					
Ventilated Improved pit latrines	1200 (53.3)	48 (4.0)	Ref		
Latrines without pit or slab	630 (28.0)	21 (3.3)	0.64 (0.36)	0.21 - 1.94	0.426
Pour flush latrines	420 (18.7)	7 (1.7)	0.31 (0.22)	0.07 - 1.28	0.105
Do school pit opening have fecal around it?					
No	1590 (70.7)	59 (3.7)	Ref		
Yes	660 (29.3)	17 (2.6)	1.28 (0.89)	0.33 - 5	0.722
Do school toilet can be accessed with flies?					
No	1500 (66.7)	59 (3.9)	Ref		
Yes	750 (33.3)	17 (2.3)	0.77 (0.49)	0.22 - 2.65	0.675

SE; standard error, CI; confidence interval.

**Table 10:** Results of univariate and multivariate mixed model effect analysis for factors associated with schistosomiasis infection.

Variable	Number of participants (%)	Infected (%)	Univariate			Multivariate		
			Odds ratio (SE)	95% CI	P value	Odds ratio (SE)	95% CI	P value
Sex								
Female	1136 (50.5)	80 (7.0)	Ref					
Male	1114 (49.5)	130 (1.7)	2 (0.34)	1.44,2.79	**< 0.001**	1.93 (0.33)	1.38,2.69	**< 0.001**
Age (years)								
10	534 (23.7)	37 (6.9)	Ref					
11	556 (24.7)	42 (7.6)	0.92(0.25)	0.55,1.55	0.758			
12	612 (27.2)	73 (11.9)	1.5 (0.37)	0.93, 2.42	0.092			
13	367 (16.3)	37 (10.1)	1.02 (0.29)	0.59, 1.77	0.942			
14	181 (8.0)	21 (11.6)	1.32 (0.44)	0.68, 2.55	0.406			
Did you receive deworming treatment with praziquantel?								
No	286 (12.7)	22 (7.7)	Ref					
Yes	1964 (87.3)	188 (9.6)	0.98 (0.32)	0.52, 1.85	0.95			
Did you bath, swim or play in nearby rivers, stream or ponds?								
No	1181 (52.5)	89 (7.5)	Ref					
Yes	1069 (47.5)	121 (11.3)	1.78 (0.36)	1.2, 2.63	**0.004**	1.65 (0.34)	1.11, 2.47	**0.013**
Did you go for fishing in nearby rivers, stream or ponds?								
No	1602 (71.2)	130 (8.1)	Ref					
Yes	648 (28.8)	80 (12.4)	1.79 (0.38)	1.18, 2.7	0.006			
Do you use improved water source?								
Yes	1710 (76.0)	151 (8.8)	Ref					
No	540 (24.0)	59 (10.9)	0.57 (0.27)	0.23, 1.43	0.23			
Time takes to get hand washing water and come back								
0 minutes	1200 (53.3)	134 (11.2)	Ref					
< 30 minutes	510 (22.7)	53 (10.4)	1.07 (0.48)	0.45, 2.59	0.873			
≥ 30 minutes	540 (24.0)	23 (4.3)	0.52 (0.26)	0.2, 1.4	0.198			
Hand washing facilities available at school								
No any handwashing facility	210 (9.3)	8 (3.8)	Ref					
Water only	1680 (74.7)	185 (11.0)	2.03 (1.56)	0.45, 9.18	0.359			
Water and soap/ashes	360 (16.0)	17 (4.7)	1.46 (1.33)	0.24, 8.77	0.68			
Type of latrine available at school								
Ventilated Improved pit latrines	1200 (53.3)	113 (9.4)	Ref					
Latrines without pit or slab	630 (28.0)	49 (7.8)	0.52 (0.23)	0.21, 1.24	0.137			
Pour flush latrines	420 (18.7)	48 (11.4)	0.77 (0.38)	0.29, 2.05	0.605			
Do school pit openings have fecal around it?								
No	1590 (70.7)	143 (9.0)	Ref					
Yes	660 (29.3)	67 (10.2)	0.84 (0.38)	0.35, 2.03	0.697			
Do school toilet can be accessed with flies								
No	1500 (66.7)	142 (9.5)	Ref					
Yes	750 (33.3)	68 (9.1)	0.63 (0.27)	0.27, 1.46	0.277			

## 4. Discussion

This study investigated the prevalence of STH and schistosomiasis and associated factors among SAC in five districts following multiple rounds of PC in Tanzania. A sample consisting of 2250 SAC was analysed, revealing an overall prevalence of 3.4% for STH, 1.2% for *S. mansoni* and 8.3% for *S. haematobium*. The ward level prevalence indicated that the prevalence of schistosomiasis has decreased in four districts except in Nyamba DC. Despite the decreased in prevalence of schistosomiasis and STH, ongoing monitoring and control efforts remain essential, as low prevalence can sometimes conceal localized outbreaks. In contrast, the higher prevalence of 8.3% for *S. haematobium* points to a more substantial public health challenge in these areas, emphasizing the need for targeted interventions and community awareness campaigns, especially in districts with frequent water contact activities. These findings support the view that STH and schistosomiasis remain persistent issues in Tanzania, underscoring the necessity for continued, focused intervention efforts.

Our findings demonstrate a marked decline in schistosomiasis prevalence compared to the baseline survey conducted in 2004, in which infections were identified using both hematuria (blood in urine) and the Kato-Katz technique, with prevalence rates ranging from 12.7% to 87.6% in some wards [23]. When compared to the 2021 impact assessment conducted in 29 councils within the Lake Zone which reported a prevalence of 15.8% our findings reflect a lower prevalence [24]. However, compared to the 2022 impact assessment carried out in 53 councils across the Lake, Northern, Eastern, and Southern Highland zones, our results indicate a higher prevalence [25]. These differences may be explained by the geographical variation in the study areas, with the Lake Zone historically exhibiting the highest schistosomiasis prevalence in the country [5]. There is no baseline data for STH infections, as the entire country was previously classified as highly endemic based on hospital records [26]. However, the low prevalence observed in our study suggests a decline in STH endemicity in Tanzania. Our prevalence is slightly lower than that reported in the 2021 impact assessment survey (9.2%), but higher than the 2022 survey, which reported a prevalence of 3.3%.

The WHO recommends several PC strategies based on the prevalence data obtained from impact assessment surveys for STH. For areas with a prevalence of less than 2%, WHO advises event-based PC, which may include activities such as PC during immunization for pre-SAC, at school enrollment and graduation for SAC, and during antenatal care visits for WRA [17]. Our findings show that only Iringa DC meets this WHO recommendation. Additionally, WHO recommends administering PC every two years for all at-risk groups in areas with a prevalence between 2% and less than 10% [17]. Based on our study, all four other districts fall into this category.

In case of schistosomiasis in endemic areas, when the prevalence of *Schistosoma* infection is < 10%, WHO suggests to maintain or reduce frequency (one round every 2–3 years) of PC based on historical evidence such as baseline prevalence and transmission factors. One of two approaches based on programmatic objectives and resources: (i) where there has been a program of regular PC, to continue the intervention at the same or reduced frequency towards interruption of transmission; or (ii) where there has not been a program of regular PC, to use a clinical approach of test and-treat, instead of PC targeting a population. Also, it suggests that in endemic communities with prevalence of *Schistosoma* infection ≥ 10% that demonstrate lack of an appropriate response to annual PC, despite adequate treatment coverage (≥ 75%), the program to consider biannual instead of annual PC [13]. The findings of this survey indicates that 52 wards with prevalence of < 10% within all districts require the program to continue the intervention at the reduced frequency towards interruption of transmission. For the remaining 23 wards with prevalence of ≥ 10% demonstrate lack of an appropriate response to annual PC, despite effective PC (coverage ≥ 75%), therefore the program should consider biannual instead of annual PC.

The findings from this study reveal that a range of factors, classified into child-related and water-contact activities, are associated with the prevalence of STH and schistosomiasis infections among SAC in five districts of Tanzania. Among child-related factors, gender emerged as significantly associated with the prevalence of schistosomiasis, indicating potential biological or behavioral differences in exposure or susceptibility. Water-contact activities, such as bathing, swimming, or playing in nearby rivers, streams, or ponds, were also significantly linked to schistosomiasis infection, highlighting the role of environmental exposure in transmission. While the multivariable analysis did not identify any variables significantly associated with STH infections, water-contact activities showed an association in the univariable analysis. Together, these findings more specifically indicate that the likelihood of SAC experiencing STH or schistosomiasis infection is associated with the male gender, and bathing, swimming or playing in nearby rivers, streams, or ponds. These results underscore the importance of considering both individual and environmental factors when designing targeted interventions for STH and schistosomiasis control in endemic regions.

The finding that male SAC have increased odds of schistosomiasis infection compared to females SAC is consistent with several previous studies conducted in Africa and other regions of the world [27–30]. This observation may be attributed to behavioral differences, re infection post treatment, or noncompliance with treatment. male children are often more likely to engage in activities involving water contact, such as swimming, fishing, or playing in rivers, streams, and ponds. These activities increase their exposure to water sources where schistosomiasis-transmitting snails are commonly found [31]. Although females have some exposure activities which are particular to them, such as household chores, like washing dishes and doing laundry in contaminated water bodies, this seems to not be enough to increase their probability of infection but their water contact activities involve the use of soap which may have a cancericidal effect and thus reduce their risk whilst exposed to contaminated water [32]. These findings highlight the need of tailored health education campaigns to address gender-specific risks, focusing on increasing awareness among boys and their caregivers about the dangers of water-contact activities in areas where schistosomiasis is endemic.

Consistent with outcomes in previous studies [33,34], the present study found that SCA who were engaged in swimming, bathing or playing on water bodies are more likely to get infected than those who don’t. A logical explanation for these findings is that schistosomiasis is commonly transmitted through contact with freshwater contaminated by certain types of snails that release infectious larvae (cercariae) into the water. When children come into contact with contaminated water, these larvae can penetrate their skin, leading to infection. SAC often spend time in or near natural water sources for recreation or daily hygiene, making them more vulnerable to infection. Public health interventions, therefore, must address not only individual health practices but also broader access to safe water, safe recreational options, and health education to reduce the prevalence of schistosomiasis in endemic areas. Contrary to our findings, a study done by [35] in South Africa found a low prevalence of infection among children who swim compared to their counterparts. This increased infection risk among children engaging in these water-contact activities emphasizes the importance of understanding behavioral and environmental factors in schistosomiasis transmission. Public health interventions, therefore, must address not only individual health practices but also broader access to safe water, safe recreational options, and health education to reduce the prevalence of schistosomiasis in endemic areas.

## 5. Conclusion

This study examined the prevalence of STH and schistosomiasis and associated risk factors among SAC across five districts in Tanzania following multiple rounds of PC. The low prevalence of STH (3.4%) and *S. mansoni* (1.2%) infections suggests that PC interventions have effectively reduced these infections in the target populations. However, the higher prevalence of S. haematobium (8.3%) presents a notable public health challenge, emphasizing the need for ongoing targeted interventions and community awareness, especially in districts with frequent water-contact activities. The findings indicate that both child-specific and environmental factors, such as male gender and water-contact activities, are linked to schistosomiasis prevalence, underscoring the need for interventions that address these aspects to control STH and schistosomiasis in endemic regions.

### 5.1. Limitations

This study has certain limitations that may influence the generalizability and interpretation of the findings. First, the use of a single sample collection restricts the ability to observe fluctuations in infection prevalences over time. Second, this study was the cross-sectional study and data was gathered at a single point in time, limiting causal inferences and only capturing associations rather than cause-effect relationships. Additionally, while the study focused on childhood factors such as gender and water-contact activities, it did not account for other potential environmental household-level factors and caregivers’ factors that could influence STH and schistosomiasis transmission.
